# Supplementary material for: Time-frequency super-resolution with superlets
Source: Nat Commun. 2021 Jan 12;12:337. doi: 10.1038/s41467-020-20539-9 (PMC7803992; doi:10.1038/s41467-020-20539-9)
Supplement: Supplementary file 1 — Supplementary Information [file 41467_2020_20539_MOESM1_ESM.pdf]

Supplementary Information

Time-frequency super-resolution with superlets

Vasile V. Moca, Harald Bârzan, Adriana Nagy-Dăbâcan, Raul C. Mureșan

Table of contents

I. Supplementary figures for Main Text 2

II. Fundaments of spectrograms and scalograms 5

Time-frequency versus time-scale energy distributions 5

Wavelet normalization and the interpretation of the scalogram 7

The modified "instantaneous power" scalogram 10

III. *Superlets* and redundancy suppression: towards multiscale TFRs 13

Redundancy suppression by superlets – analytical derivation 13

Constant absolute bandwidth superlets 18

Interpretation of superlets and relation to MMCE 20

IV. Resolution of TFR/TSR representations 22

The "Uncertainty Product" and its single-sided variant 22

Marginals of high-resolution representations 26

V. Gallery of examples on single trial electrophysiology data 30

VI. Supplementary Information references 33

I. Supplementary figures for Main Text

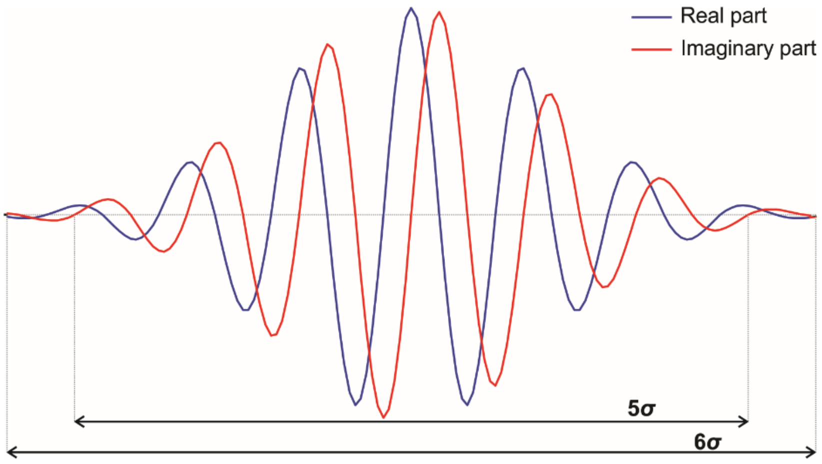

Supplementary Fig. 1 The complex Morlet wavelet. Parameters: c=6 cycles and central frequency f = 50 Hz, generated at a sampling rate of 1 kHz. The wavelet is generated such that it covers ±3 full periods (6 cycles) within ±2.5σ. The total size of the window used for convolution is always larger, encompassing 6σ.

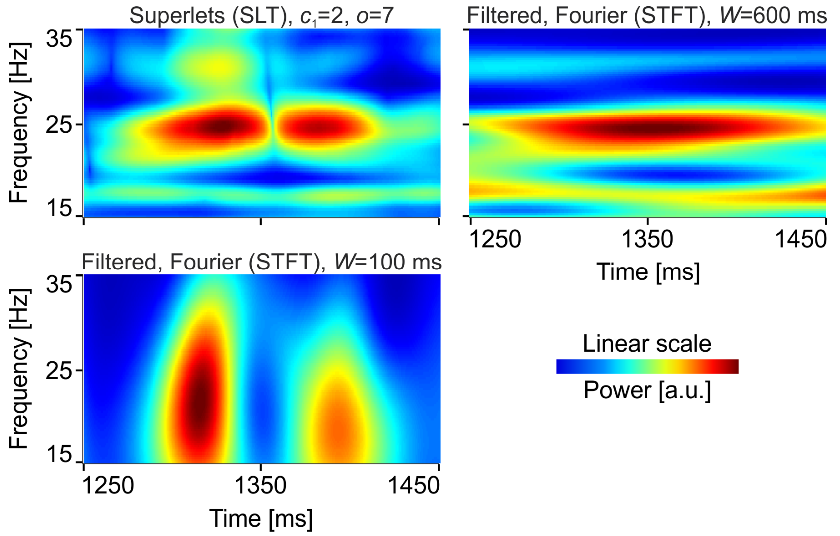

Supplementary Fig. 2 Identification of the oscillation packets revealed by super-resolution using Fourier analysis (STFT). Top-left: super-resolution using multiplicative *superlets* with c_1 = 2, and o = 7. Top-right: identification of frequency components using a

large Fourier window (W = 600 ms). Bottom-left: identification of temporal components using a small Fourier window (W = 100 ms). The signal was band-pass filtered at 10-40Hz for the STFT analysis only. See also Fig. 4e in Main Text.

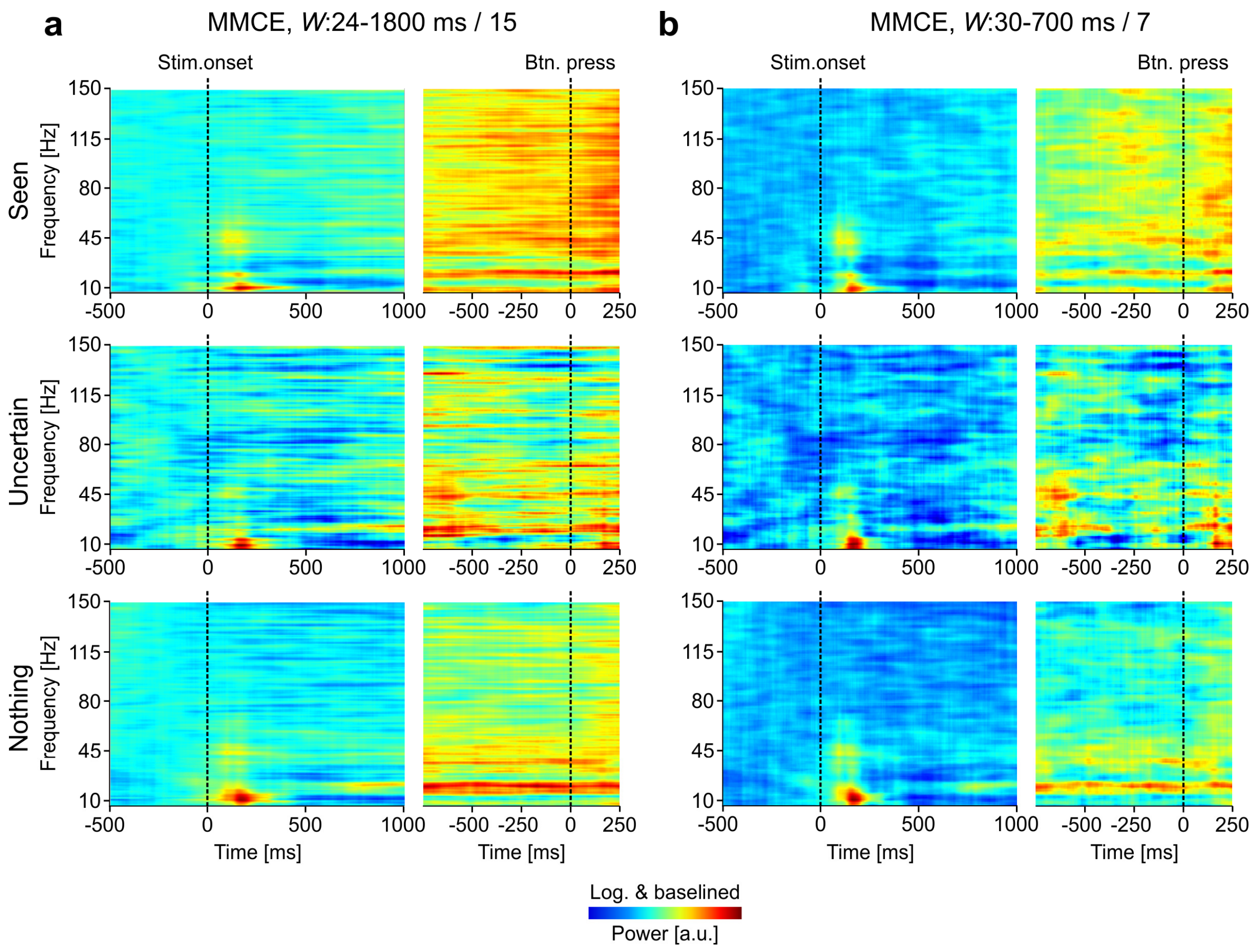

Supplementary Fig. 3 Exploration of MMCE parameters. a MMCE having window sizes matched with the extent of shortest and longest wavelets of the ASLT with orders spanning 1-15 and c_1 = 3 cycles (see Fig. 6a in Main Text). **b** MMCE with trade-off parameters (identical to Fig. 6a, shown here to facilitate comparisons).

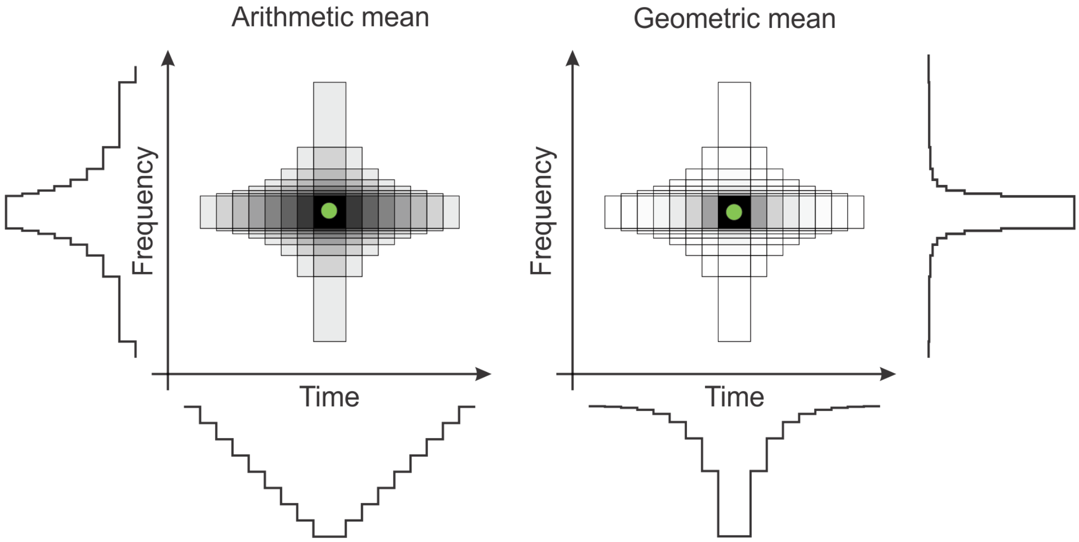

Supplementary Fig. 4 Comparison of arithmetic mean and geometric mean. Estimates using a set of windows with varying frequency and temporal resolution are combined using the arithmetic (left) or geometric (right) mean conveying different time and frequency concentration profiles. In particular, the arithmetic mean suffers from significantly higher temporal smearing than the geometric mean, thus offering a poorer temporal resolution.

II. Fundaments of spectrograms and scalograms

Time-frequency versus time-scale energy distributions

In signal processing, it is often useful to create a time-frequency representation (TFR) of a signal that reflects how the signal's energy is dispersed over time and frequency. Among the most popular TFRs is the *spectrogram*, which is the squared modulus of the short-time Fourier transform (STFT):

$$\mathrm{STFT}_x(t,\omega) = \frac{1}{\sqrt{2\pi}}\int_{-\infty}^{\infty} x(\tau)h^*(\tau - t)e^{-j\omega\tau}d\tau \qquad (s1)$$

$$S_x(t,\omega) = |\mathrm{STFT}(t,\omega)|^2 \qquad (s2)$$

where, h is a time-localizing window function. If $|h|^2$ integrates to unity (unit energy[1,2]), then the following holds:

$$\int_{-\infty}^{\infty}\int_{-\infty}^{\infty} S_x(t,\omega)dtd\omega = E_x \qquad (s3)$$

$$E_x = \int_{-\infty}^{\infty} |x(t)|^2 dt \qquad (s4)$$

where, E_x is the energy of the signal. While conserving the energy of the signal, the spectrogram is rather poor at representing the signal's instantaneous frequency and it is plagued by a trade-off between temporal and frequency resolution due to the Gabor-Heisenberg uncertainty principle[3].

The spectrogram represents the amount of energy in the differential element $\Delta t \Delta \omega$ at location (t,ω) in the time-frequency plane[4]. As we shall see below, there is a fundamental difference in interpretation for the case of time-scale representations.

By contrast to TFRs, time-scale representations (TSR) are obtained by a scaling operation in time rather than frequency "shifting" like in TFRs[5]. A TSR can be obtained using the continuous wavelet transform (CWT), which is a convolution of the signal with a family of functions localized in both time and frequency, called wavelets, ψ, and which are scaled versions of a "mother wavelet", ψ_m:

$$\psi(t,a) = \frac{1}{\sqrt{a}}\psi_m\left(\frac{t}{a}\right) \qquad (s5)$$

$$\mathrm{CWT}_x(t,a) = \int_{-\infty}^{\infty} x(\tau)\, \psi^*(\tau - t) d\tau = \frac{1}{\sqrt{a}} \int_{-\infty}^{\infty} x(\tau)\, \psi_m^* \left(\frac{\tau - t}{a}\right) d\tau \qquad (s6)$$

For the CWT to be invertible, a so-called "admissibility" condition has to be obeyed by the mother wavelet[6]:

$$C_m = \int_{-\infty}^{\infty} |F\{\psi_m\}(\omega)|^2 \frac{d\omega}{|\omega|} < \infty \qquad (s7)$$

where, $F\{\psi_m\}$ is the Fourier transform of the mother wavelet. Eq. (s7) entails that the mother wavelet is the impulse response of a band-pass filter, oscillating in the time domain[7], hence the name wavelet. C_m is called the "admissibility constant" and depends on the choice of the mother wavelet. For simplicity, let us consider that the mother wavelet is normalized such that $C_m = 1$.

Because wavelets are band-pass filters, one can express the CWT as a function of frequency by scaling the central frequency of the mother wavelet, ω_m:

$$a = \frac{\omega_m}{\omega} \qquad (s8)$$

Therefore, the CWT corresponds to the application of a constant-Q filter bank[7].

The *scalogram*[5] is a TSR obtained from the CWT by simply the squaring its modulus:

$$T_x(t,a) = |\mathrm{CWT}_x(t,a)|^2 \qquad (s9)$$

Importantly, unlike the case of the spectrogram [eq. (s3)] the energy of the signal cannot be simply recovered from the integrated scalogram, but an extra scaling term has to be considered[7,8]:

$$\int_{-\infty}^{\infty} \int_{-\infty}^{\infty} T_x(t,a) \frac{dt da}{a^2} = E_x \qquad (s10)$$

Unlike the spectrogram, **the scalogram at (*t*,*a*) represents the amount of energy in the differential** $\frac{\Delta t \Delta a}{a^2}$, i.e. it estimates the energy within the window of the wavelet and spread through its equivalent filter's bandwidth. As the wavelet dilates, the effective bandwidth of the band-pass filter shrinks, i.e. as the time window expands the frequency band shrinks (see also Fig. 1 in Main Text and the paper of Rioul and Flandrin, 1992[7]). **This has the result that the "observed" energy at location (*t*,*a*) in the scalogram becomes proportional to the squared scale**. Alternatively, in a frequency-based representation, as

frequency increases, the energy becomes increasingly spread out as time shrinks and the bandwidth of the wavelet's equivalent band-pass filter increases. This has very important implications, requiring special attention on wavelet normalization, as we will discuss below.

Wavelet normalization and the interpretation of the scalogram

For a plane sine wave of frequency ω_s (tone) and much longer than the window, the instantaneous energy density estimate at (t,ω_s) in the spectrogram is the same, irrespective of the tone's frequency (Supplementary Fig. 5a). This is desirable in TFRs, which attempt to represent the instantaneous energy/fractional energy density at the instantaneous frequency at a particular time instant.

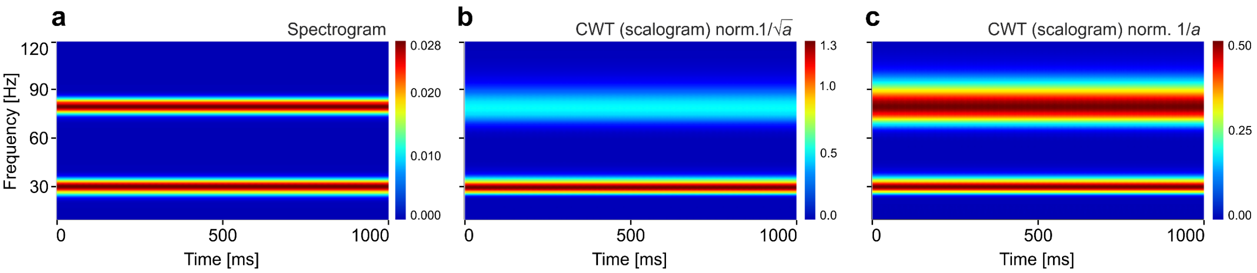

Supplementary Fig. 5 Representation of power in spectrograms and scalograms for two tones at 30 and 80 Hz. a Spectrogram. **b** Traditional scalogram with $1/\sqrt{a}$ normalization. **c** Modified scalogram with $1/a$ normalization. Note the power scale correspondence between **b** and **c**: the power at 80 Hz is identical in the two scalograms. Absolute power shown (linear scale).

Unfortunately, the scalogram does not share this nice property of the spectrogram. Considering the previous example, as the frequency of a tone increases [note that we are operating in frequency now, i.e. inverse of scale; see eq. (s8)], the scalogram at (t,ω_s) shows a decreasing peak energy, i.e. the power of the tone is spread out in increasingly larger frequency bands around ω_s, as the latter increases. The net effect of this is that "power" in the scalogram representation becomes progressively diluted/spread as frequency

increases [or progressively concentrated as frequency decreases; see eq. (s10)]. Therefore, two tones with identical magnitudes but different frequency will appear "different" in the scalogram, as the higher frequency is diluted relative to the lower one (see Supplementary Fig. 5b). The energy density of each sine wave can only be recovered by integrating across the effective bandwidth of the wavelets' having central frequencies that match the sines' frequencies. This phenomenon renders traditional scalograms fundamentally different from what TFR representations intuitively show.

The reason for this "dilution" in the scalogram pertains to the normalization of the wavelets when scaling the mother wavelet. Let us revisit eq. (s5). One can show that wavelets are normalized such that each wavelet in the family has the same energy as the mother wavelet:

$$\psi(t,a) = \frac{1}{\sqrt{a}}\psi_m\left(\frac{t}{a}\right) \;\Rightarrow\; \int_{-\infty}^{\infty} |\psi(t,a)|^2 dt = \int_{-\infty}^{\infty} \left|\frac{1}{\sqrt{a}}\psi_m\left(\frac{t}{a}\right)\right|^2 dt \qquad (s11)$$

With a change of variables:

$$u = \frac{t}{a} \;\Rightarrow\; t = a\,u \;\Rightarrow\; dt = a\,du \qquad (s12)$$

we have:

$$\int_{-\infty}^{\infty} \left|\frac{1}{\sqrt{a}}\psi_m\left(\frac{t}{a}\right)\right|^2 dt = \frac{1}{a}\int_{-\infty}^{\infty} |\psi_m(u)|^2 a\,du = \int_{-\infty}^{\infty} |\psi_m(u)|^2 du \qquad (s13)$$

hence the energy of the "child" wavelet is the same as that of the mother wavelet:

$$\int_{-\infty}^{\infty} |\psi(t,a)|^2 dt = \int_{-\infty}^{\infty} |\psi_m(t)|^2 dt \qquad (s14)$$

In the following, we will consider a Morlet wavelet of unit energy and demonstrate that the scalogram of a single, infinitely long tone dilutes/concentrates the instantaneous power estimate of the wave with a factor that is proportional to the wave's frequency/period. Let us consider the analytic modified Morlet (Gabor) wavelet[9]:

$$\psi_m(t) = \pi^{-\frac{1}{4}}\, e^{-\frac{t^2}{2}}\, e^{j\omega_m t} \qquad (s15)$$

It can be easily shown that this wavelet has unit energy. For $\omega_m = 6$, this mother wavelet is admissible. Its Fourier transform is:

$$\hat{\psi}_m(a\omega) = \pi^{-\frac{1}{4}} H(\omega)\, e^{-\frac{(a\omega-\omega_m)^2}{2}} \quad (s16)$$

where, $H(\omega)$ is the Heaviside step function. The latter reflects the fact that the wavelet's Fourier transform is a Gaussian shifted to ω_m in the positive part of the frequency spectrum (since ω_m is positive and should be large enough to render the mother wavelet admissible). Indeed, due to the admissibility criterion one can neglect the negative side of this wavelet's spectrum[10]. This observation will be very important later for normalization purposes.

Let us consider an infinitely long plane tone with frequency ω_s, defined as:

$$x_{\sin}(t) = A\sin(\omega_s t) \quad (s17)$$

By replacing the scale with frequency, as defined in eq. (s8), and considering eq. (s6), the energy (power) of $x_{\sin}$ estimated by the scalogram at frequency ω_s becomes:

$$T_{x_{\sin}}(t,\omega_s) = \left|\mathrm{CWT}_{x_{\sin}}(t,\omega_s)\right|^2 = \left|\sqrt{\frac{\omega_s}{\omega_m}}\int_{-\infty}^{\infty} x_{\sin}(\tau)\, \psi_m^*\left(\frac{\omega_s}{\omega_m}[\tau - t]\right) d\tau\right|^2 = |I(t)|^2 \quad (s18)$$

We can resolve the right-hand integral, $I(t)$, by expressing the convolution in the time-domain as the inverse Fourier transform of a multiplication in the frequency-domain. The latter involves the Fourier transforms of the signal and wavelet[7,11]:

$$I(t) = \sqrt{\frac{\omega_s}{\omega_m}}\int_{-\infty}^{\infty} x_{\sin}(\tau)\, \psi_m^*\left(\frac{\omega_s}{\omega_m}[\tau - t]\right) d\tau = \sqrt{\frac{\omega_m}{\omega_s}}\int_{-\infty}^{\infty} \hat{x}_{sin}(\omega)\, e^{j\omega t}\, \hat{\psi}_m^*\left(\frac{\omega_m}{\omega_s}\omega\right) d\omega \quad (s19)$$

The Fourier transform of the sine wave is:

$$\hat{x}_{\sin}(\omega) = A\sqrt{2\pi}\,\frac{\delta(\omega-\omega_s) - \delta(\omega+\omega_s)}{2j} \quad (s20)$$

where, δ is the Dirac delta function. It follows from eq. (s18), (s19), and (s20) that:

$$I(t) = \frac{A\sqrt{2\pi}}{2j}\sqrt{\frac{\omega_m}{\omega_s}}\left[\hat{\psi}_m^*(\omega_m) - \hat{\psi}_m^*(-\omega_m)\right]e^{j\omega_s t} \quad (s21)$$

$$T_{x_{\sin}}(t,\omega_s) = |I(t)|^2 = A^2\,\frac{\pi}{2}\,\frac{\omega_m}{\omega_s}\left|\hat{\psi}_m^*(\omega_m) - \hat{\psi}_m^*(-\omega_m)\right|^2 \quad (s22)$$

For the unit energy Morlet wavelet in eq. (s15), considering its Fourier transform in eq. (s16), and noticing that $\omega_m > 0$, we obtain:

$$T_{\mathrm{Morlet}[x_{\sin}]}(t,\omega_s) = A^2\,\frac{\pi}{2}\,\frac{\omega_m}{\omega_s}\,\left|\hat{\psi}_m^*(\omega_m)\right|^2 = A^2\,\frac{\pi}{2}\,\frac{\omega_m}{\omega_s}\,\pi^{-\frac{1}{2}} = A^2\,\frac{\sqrt{\pi}}{2}\,\frac{\omega_m}{\omega_s} \qquad (s23)$$

The instantaneous power of the tone is $A^2/2$, but **the Morlet scalogram at the frequency of the wave produces an estimate which is dependent on frequency**: the larger the frequency of the tone, the more "diluted" its representation, and, *vice versa*, the lower the tone frequency the more concentrated its representation (see Supplementary Fig. 5b). This last result is very important because it demonstrates that the scalogram, in its original form, does not provide the same qualitative results as the spectrogram and, therefore, its interpretation is fundamentally different.

The modified "instantaneous power" scalogram

It has been realized for a while now that the scalogram, in its original form, is not very useful for a TFR-like representation of a signal. In fact, the standard CWT is rarely used – most wavelet-based representations involve a dyadic sequence (e.g., see the Discrete Wavelet Transform[12]) where the mother wavelet is scaled by powers of 2. This can be viewed as a sequence of filters which recover the energy of the signal from bands whose central frequencies are powers of 2.

As early as 1992, Mallat and Zhong[12] used a different kind of wavelet normalization, in the context of DWT: the wavelet was normalized to the scale instead of the latter's square root. In the same year, Delprat et al.[13] have shown that the same normalization is useful for ridge detection using wavelets[14,15]. The usefulness of this normalization was rediscovered later in the context of the scalogram, especially by scientists in Geophysics[16–20]. Moreover, some of the most popular signal analysis tools, e.g., MATLAB use this normalization implicitly[21].

We argue that wavelet normalization is often ignored and receives too little attention, in spite of having a dramatic impact on what its corresponding scalogram represents. In principle one would like to keep the benefits of multiscale (multiresolution), offered by wavelets, while also being able to recover the instantaneous power of signal components. As we will show below, the normalization to the scale, a, provides a series of advantages by correctly estimating instantaneous power of tones at

different frequencies.

The traditional wavelet normalization in eq. (s5) is said to be an L^2 norm because it preserves energy. The new normalization is L^1 norm, as it preserves amplitude (integrated modulus)[16,17], and is defined as:

$$\psi(t,a) = \frac{1}{a}\psi_m\left(\frac{t}{a}\right) \qquad (s24)$$

To avoid confusion, we will refer to the scalogram that relies on the CWT with the modified wavelet normalization (CWTM), as N_x:

$$N_x(t,a) = |\mathrm{CWTM}_x(t,a)|^2 = \left|\frac{1}{a}\int_{-\infty}^{\infty} x(\tau)\,\psi_m^*\left(\frac{\tau-t}{a}\right)d\tau\right|^2 \qquad (s25)$$

Following the same procedure as before, we can show that **the wavelets of this type are normalized such that each wavelet in the family has the same integrated modulus** as the mother wavelet:

$$\psi(t,a) = \frac{1}{a}\psi_m\left(\frac{t}{a}\right) \;\Rightarrow\; \int_{-\infty}^{\infty}|\psi(t,a)|dt = \int_{-\infty}^{\infty}\left|\frac{1}{a}\psi_m\left(\frac{t}{a}\right)\right| dt \qquad (s26)$$

Using the same change of variables:

$$u = \frac{t}{a} \;\Rightarrow\; t = a\,u \;\Rightarrow\; dt = a\,du \qquad (s27)$$

we have:

$$\int_{-\infty}^{\infty}\left|\frac{1}{a}\psi_m\left(\frac{t}{a}\right)\right| dt = \frac{1}{a}\int_{-\infty}^{\infty}|\psi_m(u)|\;a\,du = \int_{-\infty}^{\infty}|\psi_m(u)|du \qquad (s28)$$

hence the integrated modulus of the “child” wavelet is the same as that of the mother wavelet:

$$\int_{-\infty}^{\infty}|\psi(t,a)|dt = \int_{-\infty}^{\infty}|\psi_m(t)|dt \qquad (s29)$$

The energy of the signal can be recovered from the modified scalogram using the following formula:

$$\int_{-\infty}^{\infty}\int_{-\infty}^{\infty} N_x(t,a)\frac{dtda}{a} = E_x \qquad (s30)$$

Next, following the same demonstration as above, we will consider a modified Morlet

wavelet of **unit integral**. We will show that **the modified Morlet scalogram of a single long tone converges to half the power of the wave, provided the wavelet is unit integral**. Consider the unit integral Morlet mother wavelet:

$$\psi_m(t) = \frac{1}{\sqrt{2\pi}}\, e^{-\frac{t^2}{2}}\, e^{j\omega_m t} \qquad (s31)$$

with Fourier transform:

$$\hat{\psi}_m(a\omega) = \frac{1}{\sqrt{2\pi}}\, H(\omega)\, e^{-\frac{(a\omega-\omega_m)^2}{2}} \qquad (s32)$$

With the same steps as above, replacing the scale with frequency, as defined in eq. (s8), and considering eq. (s25), the power of $x_{\sin}$ estimated by the modified scalogram at frequency ω_s becomes:

$$N_{x_{\sin}}(t,\omega_s) = \left|\mathrm{CWTM}_{x_{\sin}}(t,\omega_s)\right|^2 = \left|\frac{\omega_s}{\omega_m}\int_{-\infty}^{\infty} x_{\sin}(\tau)\, \psi_m^*\left(\frac{\omega_s}{\omega_m}[\tau - t]\right) d\tau\right|^2 = |I(t)|^2 \quad (s33)$$

Again, the right-hand integral, $I(t)$, can be expressed in the frequency-domain:

$$I(t) = \frac{\omega_s}{\omega_m}\int_{-\infty}^{\infty} x_{\sin}(\tau)\, \psi_m^*\left(\frac{\omega_s}{\omega_m}[\tau - t]\right) d\tau = \int_{-\infty}^{\infty} \hat{x}_{\sin}(\omega)\, e^{j\omega t}\, \hat{\psi}_m^*\left(\frac{\omega_m}{\omega_s}\omega\right) d\omega \qquad (s34)$$

It follows from eq. (s20) and (s34) that:

$$I(t) = \frac{A\sqrt{2\pi}}{2j}\left[\hat{\psi}_m^*(\omega_m) - \hat{\psi}_m^*(-\omega_m)\right] e^{j\omega_s t} \qquad (s35)$$

$$N_{x_{\sin}}(t,\omega_s) = |I(t)|^2 = A^2\, \frac{\pi}{2}\, \left|\hat{\psi}_m^*(\omega_m) - \hat{\psi}_m^*(-\omega_m)\right|^2 \qquad (s36)$$

For the unit integral Morlet wavelet in eq. (s31) and its Fourier transform in eq. (s32), with $\omega_m > 0$, we obtain:

$$N_{\mathrm{Morlet}[x_{\sin}]}(t,\omega_s) = A^2\, \frac{\pi}{2}\, \left|\hat{\psi}_m^*(\omega_m)\right|^2 = A^2\, \frac{\pi}{2}\, \frac{1}{2\pi} = \frac{A^2}{4} \qquad (s37)$$

Thus, **the modified Morlet scalogram at the frequency of the tone gives a power estimate that is half the instantaneous power of the wave and is independent of the latter's frequency.** Considering that the Morlet wavelet only recovers power from half the spectrum, one can simply multiply the estimate by 2 to obtain the accurate instantaneous power of the tone (note that this holds for real signals only). An example modified

scalogram is shown in Supplementary Fig. 5c, contrasted with the "classical" scalogram that uses the traditional normalization (Supplementary Fig. 5b).

A comprehensive discussion of "classical", $1/\sqrt{a}$ normalization, versus modified, $1/a$ normalization, can be found in Lilly 2017[16]. When fitting a scaled and translated wavelet to a signal, wavelet normalization can be viewed from the perspective of an optimization problem that minimizes fitting error. Indeed, the maxima in a scalogram that uses "classical" energy normalization indicate the best fit parameters (shift and scale) that minimize integrated squared error between the signal and the wavelet. The problem with this fitting is that it favors optima for larger wavelets, which can harvest more energy in a signal with multiple neighboring elements—indeed, as Supplementary Fig. 5b shows, the representation becomes more concentrated for lower frequencies (longer wavelets). **Instead, the modified normalization (also called amplitude normalization), divides the energy harvested by the wavelet by its size, effectively capturing instantaneous power, or energy at scale. This provides a scale-invariant representation of instantaneous power—energy of event relative to its scale.** In the optimization framework described above, the modified normalization is equivalent to optimizing power[16].

III. *Superlets* and redundancy suppression: towards multiscale TFRs

Redundancy suppression by superlets – analytical derivation

The relative time resolution of the scalogram is excellent: it adapts the wavelet to the particular scale (multiscale or multiresolution[2,22]) to achieve outstanding temporal localization. As argued in the Main Text (see Fig. 1), by temporal localization we mean temporal resolution relative to the scale (cycles) of each frequency component.

Unfortunately, as we have seen, the multiscale advantage of wavelets comes at a cost: loss of frequency resolution with increasing frequency. The idea behind *superlets* is simple: attempt to increase frequency resolution in the upper frequencies by using longer wavelets, while preserving the good temporal resolution of short wavelets. Wavelets are band-pass filters and longer wavelets (more cycles) have narrower bands. To reduce redundancy in the representation, the idea of *superlets* is to "filter" the broad frequency

response of short wavelets with narrow band-pass "filters" of longer wavelets.

We will next determine analytically how the *superlet* achieves frequency concentration compared to single wavelets. For the sake of consistency with the other proofs, we will operate with angular frequency, ω. Let us first consider the Morlet wavelet in eq. (1) from the Main Text, and write its mother wavelet in angular frequency form:

$$\psi_m(t) = \frac{1}{B_{c_m}\sqrt{2\pi}} e^{-\frac{t^2}{2{B_{c_m}}^2}} e^{j\omega_m t} \tag{s38}$$

$$B_{c_m} = \frac{2\pi c_m}{5\omega_m} \tag{s39}$$

where, we fixed k_{sd} to 5.

The number of cycles c_m is fixed for a mother wavelet and should not be chosen too small in order to render the wavelet admissible. On the other hand, for this particular formulation of the Morlet wavelet, ω_m is arbitrary and, without any loss of generality, it can be set to 1. The mother wavelet then becomes:

$$\psi_m(t) = \frac{5\omega_m}{c_m(2\pi)^{3/2}} e^{-\frac{1}{2}\left[\frac{5\omega_m}{2\pi c_m}\right]^2} e^{j\omega_m t} = \frac{5}{c_m(2\pi)^{3/2}} e^{-\frac{1}{2}\left[\frac{5t}{2\pi c_m}\right]^2} e^{jt} \tag{s40}$$

The Fourier transform of the mother wavelet is given by:

$$\hat{\psi}_m(a\omega) = \frac{1}{\sqrt{2\pi}} H(\omega)\, e^{-\frac{(a\omega-1)^2}{2}\cdot\left[\frac{2\pi c_m}{5}\right]^2} \tag{s41}$$

Noticing that the scale can be expressed as $a = \omega_m/\omega = 1/\omega$ (eq. s8), the formula of the child wavelet can be written according to (s24) as:

$$\psi(t,\omega) = \frac{5\omega}{c_m(2\pi)^{3/2}} e^{-\frac{1}{2}\left[\frac{5\omega t}{2\pi c_m}\right]^2} e^{j\omega t} \tag{s42}$$

The Morlet wavelet used in *superlets* is scaled according to the modified CWT. It can be easily checked that this wavelet has unit integrated modulus.

Using the definition of the *superlet* from eqs. (3-5) in the Main Text, we can define a multiplicative *superlets* scalogram as:

$$L_{x,c_1,o}(t,a) = \prod_{i=1}^{o} \left| \sqrt{2}\frac{1}{a}\int_{-\infty}^{\infty} x(\tau)\, \psi_i^*\left(\frac{\tau-t}{a}\right) d\tau \right|^{\frac{2}{o}} \tag{s43}$$

$$\psi_i(t) = \frac{5}{c_1 \cdot i \cdot (2\pi)^{3/2}} e^{-\frac{1}{2}\left[\frac{5t}{2\pi c_1 \cdot i}\right]^2} e^{jt} \qquad (s44)$$

where, $\psi_i(t)$ is i-th mother wavelet in the set, o is the order and c_1 is the number of base wavelet cycles. In other words, the *superlets* scalogram is the geometric mean of modified scalograms with mother wavelets having increasingly higher number of cycles $(c_1 \cdot i)$. **Importantly, all results in the Main Text and the Supplementary Information pertaining to the SLT depict *superlets* scalograms.**

As mentioned above, the aim of the *superlet* is to reduce the redundancy of the representation in the higher frequency bands. Because the *superlet* is a geometric combination of individual band-pass filters (wavelets), it is itself also a band-pass filter. To determine how the *superlet* reduces redundancy, we will next analytically compute the "band-pass" characteristics of the *superlet* and compare it to the band-pass characteristics of the shortest and longest wavelets in its set.

The first step is to compute the response of a child Morlet wavelet with central frequency ω_a to a tone of arbitrary frequency $\omega_s \neq \omega_a$. Taking a similar route like in eq. (s33-s37), we have:

$$N_{x_{\sin}}(t, \omega_a) = \left| \int_{-\infty}^{\infty} \hat{x}_{sin}(\omega)\, e^{j\omega t}\, \hat{\psi}_m^*\left(\frac{\omega_m}{\omega_a}\omega\right) d\omega \right|^2 \qquad (s45)$$

Since we fixed ω_m to 1, we obtain:

$$N_{x_{\sin}}(t, \omega_a) = \left| \frac{A\sqrt{2\pi}}{2j} \left[\hat{\psi}_m^*\left(\frac{\omega_s}{\omega_a}\right) - \hat{\psi}_m^*\left(-\frac{\omega_s}{\omega_a}\right) \right] e^{j\omega_s t} \right|^2 \qquad (s46)$$

For the unit integral Morlet wavelet we obtain:

$$N_{\mathrm{Morlet}[x_{\sin}]}(t, \omega_a) = A^2\, \frac{\pi}{2} \left| \hat{\psi}_m^*\left(\frac{\omega_s}{\omega_a}\right) \right|^2 \qquad (s47)$$

Thus:

$$N_{\mathrm{Morlet}[x_{\sin}]}(t, \omega_a) = \frac{A^2}{4}\, e^{-\frac{1}{25} 4\pi^2 c_m{}^2 \left(\frac{\omega_s}{\omega_a} - 1\right)^2} \qquad (s48)$$

Equation (s48) is very important as, together with eq. (s43), it allows us to compute the response of the *superlet* to a tone with arbitrary frequency, ω_s, as a function of the central frequency of its component wavelets, ω_a, and the base cycles of their mother wavelets, c_i:

$$L_{x,c_1,o}(t,\omega_a) = \frac{A^2}{2}\prod_{i=1}^{o}\left[e^{-\frac{1}{25}4\pi^2 i^2 {c_1}^2\left(\frac{\omega_s}{\omega_a}-1\right)^2}\right]^{\frac{1}{o}} \qquad (s49)$$

$$L_{x,c_1,o}(t,\omega_a) = \frac{A^2}{2}\, e^{-\frac{1}{25}4\pi^2 {c_1}^2\left(\frac{\omega_s}{\omega_a}-1\right)^2 \frac{\sum_{i=1}^{o} i^2}{o}} \qquad (s50)$$

We finally obtain the response of the *superlet* to a tone of arbitrary frequency, as:

$$L_{x,c_1,o}(t,\omega_a) = \frac{A^2}{2}\, e^{-\frac{1}{25}4\pi^2 {c_1}^2\left(\frac{\omega_s}{\omega_a}-1\right)^2 \frac{(o+1)(2o+1)}{6}} \qquad (s51)$$

When o=1, eq. (s51) becomes equivalent to the single wavelet case in eq. (s48), multiplied by the power correction coefficient, 2, of the superlet. It is also evident that when the frequency of the tone matches the central frequency of the *superlet*, we recover the power of the tone:

$$L_{x,c_1,o}(t,\omega_a=\omega_s) = \frac{A^2}{2} \qquad (s52)$$

Notably, just like in the case of modified wavelets, the *superlet's* response to a tone located at its central frequency does not depend on the exact frequency of the tone. Moreover, the *superlet* recovers the correct instantaneous power of the tone.

However, as soon as the central frequency of the *superlet* departs from the frequency of the tone, the former's response is rapidly dampened:

$$L_{x,c_1,o}(t,\omega_s+\Delta\omega) = \frac{A^2}{2}\, e^{-\frac{1}{25}4\pi^2 {c_1}^2\left(\frac{\Delta\omega}{\omega_s+\Delta\omega}\right)^2 \frac{(o+1)(2\cdot o+1)}{6}} \qquad (s53)$$

Let us define the "*superlet* dampening factor", as:

$$SDF_{c_1,o}\left(\frac{\Delta\omega}{\omega_s}\right) = e^{-\frac{1}{25}4\pi^2 {c_1}^2\left[\left(\frac{\Delta\omega}{\omega_s}\right)/\left(1+\left(\frac{\Delta\omega}{\omega_s}\right)\right)\right]^2 \frac{(o+1)(2\cdot o+1)}{6}} \qquad (s54)$$

and the "*superlet* outband rejection", as:

$$SOR_{c_1,o}\left(\frac{\Delta\omega}{\omega_s}\right) = 10\, log_{10}\left[SDF_{c_1,o}\left(\frac{\Delta\omega}{\omega_s}\right)\right] \qquad (s55)$$

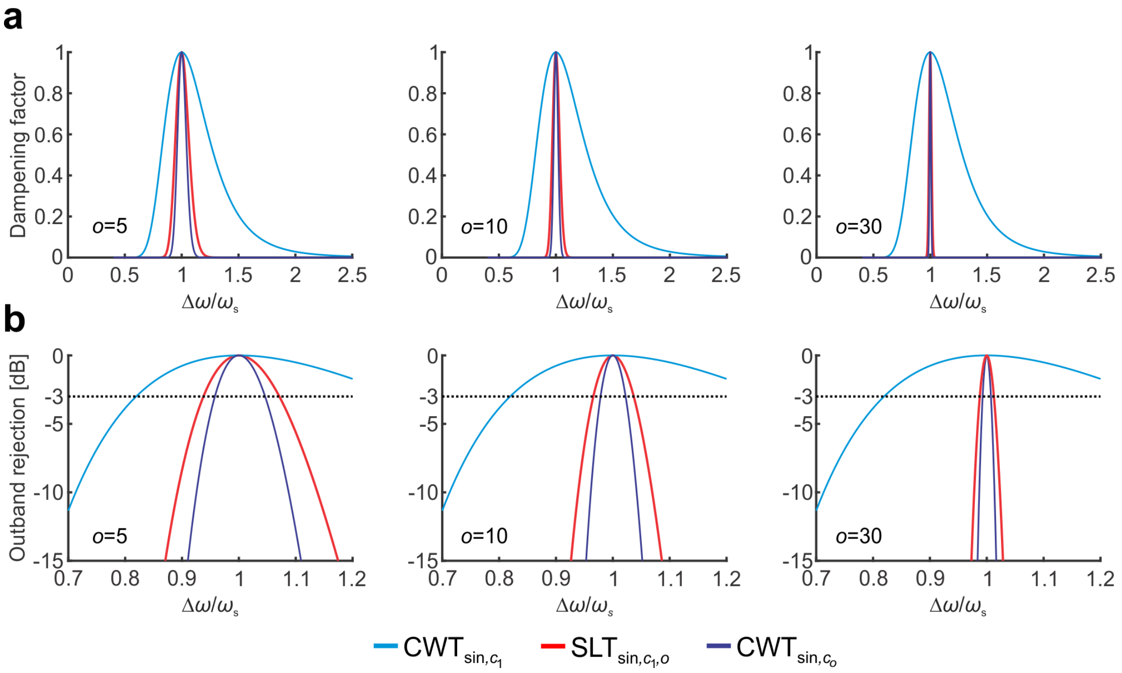

Supplementary Fig. 6 Dampening factor and outband rejection for the *superlet* (red) and the shortest (blue) and longest (dark blue) wavelets. a Dampening factor as a function of the order, o. **b** Outband rejection corresponding to **a**. c_1 = 3. Note the abscissa scale difference between **a** and **b**.

The *SDF* is dampening the response of the *superlet* exponentially fast, depending on c_1, $\frac{\Delta\omega}{\omega_s}$, and the order o. We next compared the *SDF* (Supplementary Fig. 6a) and *SOR* (Supplementary Fig. 6b) for various orders of the *superlet* to the dampening and outband rejection (computed by fixing o to 1 and setting c_1 to the number of cycles of the wavelet) of the shortest and longest wavelets in the *superlet's* set. Results indicate that the *superlet's* band-pass characteristics are very close to that of the longest wavelet in the set (maximum frequency concentration). In addition, as the order increases the *superlet* becomes increasingly sharp in frequency, i.e. its response is very localized around the frequency of the tone.

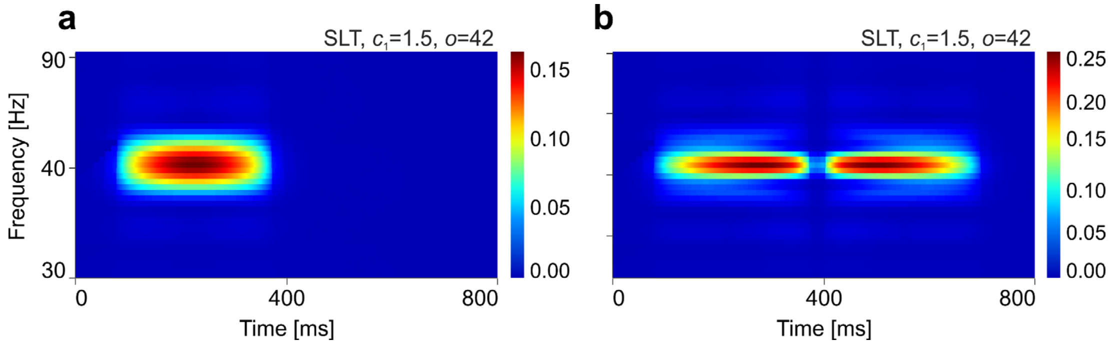

Supplementary Fig. 7 Frequency concentration of the SLT for finite sinusoidal packets. a SLT for a single packet at 40 Hz. **b** SLT on the same packet when a temporal neighbor packet is added. The signal on the right can be viewed either as two successive (time neighboring) packets or as a single sinusoidal packet interrupted by a temporal feature. Absolute power shown (linear scale).

For a tone signal, the SLT with high orders reduces redundancy drastically, concentrating energy at the frequency of the tone and correctly estimating the latter's instantaneous power. It should be noted however, that the actual gain in frequency sharpness depends on the particular signal that is being analyzed. If we are dealing with a finite, isolated packet, the *SDF* and *SOR* will depend on the particular duration of that packet: if its duration is smaller than the duration of the longest wavelet in the set, this is similar to reducing the order of the SLT. Indeed, the SLT approaches the resolution limit for a finite packet (see Fig. 1 in Main Text). Interestingly, depending on their particular time-frequency location, the presence of multiple packets in the signal can actually help sharpen the individual packets' localization (Supplementary Fig. 7). This property is shared by the MMCE method as well.

Constant absolute bandwidth superlets

The spectrogram's frequency bandwidth depends on the length of the window and is the same for each frequency (i.e., fixed time-frequency Heisenberg box[15]). As we have seen, this is not the case for the scalogram, due to its multiscale nature (the time-frequency Heisenberg box compresses in time and expands in frequency[15] as central frequency increases). The SLT compresses the frequency bandwidth of this time-frequency box in a

frequency independent manner, i.e. the relative frequency bandwidth is compressed in the same way at all frequencies (see Supplementary Fig. 6). This means that all frequencies will gain in precision, hence the "aspect ratio" of time-frequency boxes across frequencies retains its dependence on frequency, like for single wavelets.

Sometimes, it would desirable to obtain a constant frequency bandwidth while maintaining the multiscale property of the representation. This can be achieved with the adaptive SLT (ASLT). In the scalogram, the frequency resolution decreases with increase in central frequency – therefore one would need to gain more in frequency precision as the representation ascends in frequency. This is what the ASLT does: it increases the order of the *superlet* with the increase in its central frequency – see eq. (6&7) in the Main Text.

The ASLT has two additional parameters, namely the order o_{min} at the lowest frequency and the order o_{max} at the highest frequency of the representation. Here, we will show that one can calculate these parameters precisely by using eq. (s53).

Let us consider a fixed, absolute $\Delta\omega = \Delta\omega_d$. One can obtain a fixed dampening factor at $\Delta\omega_d$, irrespective of ω_s. If the tone is located at the central frequency of the *superlet* ($\omega_s = \omega_a$), we need to compute the order, o_{ASLT} required for the *superlet*, such that its dampening factor at ($\omega_a + \Delta\omega_d$) is constant. The dampening factor, d, and the frequency displacement, $\Delta\omega_d$, are fixed and are chosen by design. One then needs to solve the equation:

$$\mathrm{SDF}_{c_1,o}\left(\frac{\Delta\omega_d}{\omega_a}\right) = e^{-\frac{1}{25}4\pi^2 {c_1}^2\left[\left(\frac{\Delta\omega_d}{\omega_a}\right)\Big/\left(1+\left(\frac{\Delta\omega_d}{\omega_a}\right)\right)\right]^2\frac{(o_{\mathrm{ASLT}}+1)(2\cdot o_{\mathrm{ASLT}}+1)}{6}} = d \qquad (s56)$$

$$2{o_{\mathrm{ASLT}}}^2 + 3o_{\mathrm{ASLT}} + 1 + \frac{150\ ln\, d}{4\pi^2 {c_1}^2\left[\left(\frac{\Delta\omega_d}{\omega_a}\right)\Big/\left(1+\left(\frac{\Delta\omega_d}{\omega_a}\right)\right)\right]^2} = 0 \qquad (s57)$$

which is a quadratic equation with solutions:

$$o_{\mathrm{ASLT}} = \frac{1}{4}\left[-3 \pm \sqrt{1 - \frac{300\ ln\, d}{\pi^2 {c_1}^2\left[\left(\frac{\Delta\omega_d}{\omega_a}\right)\Big/\left(1+\left(\frac{\Delta\omega_d}{\omega_a}\right)\right)\right]^2}}\right] \qquad (s58)$$

Choosing the positive solution, we obtain the precise formula for calculating the necessary order of the ASLT, as a function of ω_a, given design parameters c_1, $\Delta\omega_d$, and d:

$$o_{\mathrm{ASLT}_{c_1,\Delta\omega_d,d}}(\omega_a) = \frac{1}{4}\left[-3 + \sqrt{1 - \frac{300\ ln\, d}{\pi^2 {c_1}^2 \left[\left(\frac{\Delta\omega_d}{\omega_a}\right) \Big/ \left(1 + \left(\frac{\Delta\omega_d}{\omega_a}\right)\right)\right]^2}}\right] \qquad (s59)$$

Interpretation of superlets and relation to MMCE

Representations constructed using the SLT are geometric combinations of modified scalograms. Therefore, these are TSRs that provide multiresolution in the wavelet sense (multiscale), while reducing representation redundancy for the higher frequencies. Due to their "non-diluting" properties, the modified scalograms have been used for wavelet ridge detection[13,15]. In some sense, the SLT behaves as a ridge detection facilitator because it concentrates the TSR in time and frequency.

Another important property of *superlets* is that the representation they provide is closer to a TFR than to a single wavelet TSR. Indeed, when the number of cycles of the wavelet is increased towards infinity, the convolution with the signal comes close to a correlation with a Fourier sine base function, where only half the power is recovered. Since the frequency bandwidth of the *superlet* is close to that of its longest wavelet (Supplementary Fig. 6), it follows that the *superlet* representation is closer to a TFR than to a scalogram computed with its base wavelet.

While sharing the same basic principle, the *superlets* and MMCE are not identical and provide different representations. The *superlets* and MMCE with Gaussian window can only be made equivalent at a single frequency, where the windows of the MMCE are matched with the sizes of the wavelets. If the wavelet transform is seen as a "windowed" spectral estimation, then one can define the CWT as a succession of spectral estimations with windows scaled as a function of frequency. It then follows that the SLT is a succession of spectral estimations with sets of windows that are *all* scaled as a function of frequency. By contrast, the MMCE uses a single set of windows to evaluate all frequencies, i.e. the window set is not scaled as a function of frequency. As a result, the SLT is a multiscale method, while the MMCE is not.

The MMCE suffers from the same problems that the traditional spectrogram suffers

from. For a spectrogram, one has to choose a window large enough to fit at least one cycle of the lowest frequency (largest period) of interest. If the oscillation packets have a constant number of cycles, their absolute duration decreases with frequency and therefore their relative size to the window decreases. Packets of higher frequency but shorter absolute duration appear “diluted” in the spectrogram. Similarly, for the MMCE, one has to choose a set of windows, but the shortest window cannot be chosen arbitrarily small because it has to obey constraints regarding the representation of the lowest frequency. As the frequency of packets with a fixed number of cycles increases, their relative duration to the durations of the windows in the MMCE set decreases, and the representation becomes diluted (Supplementary Fig. 8a).

By contrast, in *superlets* the entire set of windows is scaled with frequency and packets of equal cycle count will receive identical importance in the representation, avoiding dilution (Supplementary Fig. 8b). This kind of representation is scale-invariant, as the SLT estimates power for each scale. Indeed, the instantaneous power of a process which is self-similar across scales is the same in the SLT but not in the MMCE. Very importantly, the MMCE’s dilution cannot be alleviated by using L^1 normalization of its windows, as this is simply a global scaling factor of the entire spectrum, unlike for wavelets, where the normalization is at each individual scale.

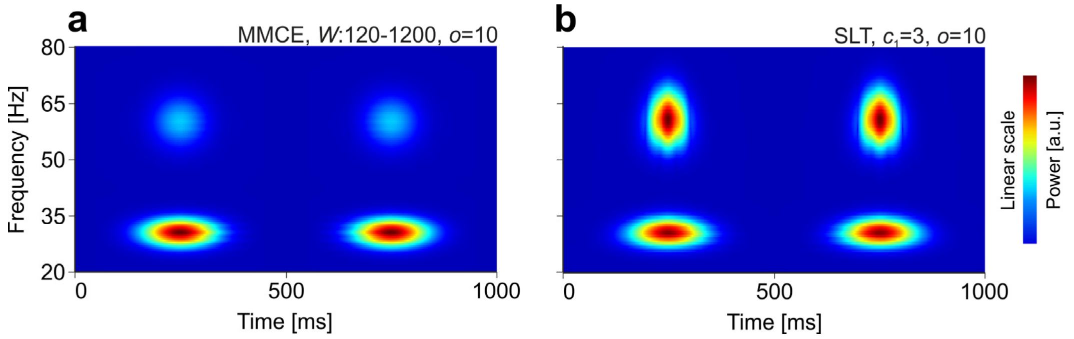

Supplementary Fig. 8 Dilution effect in MMCE. a MMCE for a signal composed of four Gaussian atoms with identical number of cycles, at two temporal locations and two frequency locations. The atoms have identical magnitude. **b** The same as in **a** but computed with *superlets*. Absolute power shown (linear scale).

From a practical point of view, it is easier for the user to choose the parameters of the SLT than for the MMCE because one operates with cycles rather than absolute time and the same base cycle parameter can be used for any range of frequencies in the representation. For the MMCE, one has to compute the sizes of the windows as a function of the frequency range of interest.

To conclude, *superlets* provide a representation that is closer to a TFR than the scalogram. They preserve the benefits of multiscale (multiresolution) while reducing the disadvantages of the original and modified scalograms. Because of the way wavelets in a *superlet* are normalized, the SLT is useful as a detection tool for biological signals, where packet durations typically decrease with increase in frequency.

IV. Resolution of TFR/TSR representations

The "Uncertainty Product" and its single-sided variant

Given a signal $x(t)$ and its time-frequency representation $TFR_x(t,\omega)$, one important question is how well the representation is localized in time and frequency compared to the signal. For the signal, a measure of localization is the uncertainty product[11], which is defined as the signal's spread in time and frequency. The spreads are defined as the standard deviations of the energy distribution over time, T, and frequency, B, and which can be computed based on the signal and its Fourier transform:

$$T^2 = \frac{1}{E_x}\int_{-\infty}^{\infty}(t - \langle t\rangle)^2 |x(t)|^2 dt \qquad (s60)$$

$$B^2 = \frac{1}{E_x}\int_{-\infty}^{\infty}(\omega - \langle \omega\rangle)^2 |\hat{x}(\omega)|^2 d\omega \qquad (s61)$$

$$\langle t\rangle = \frac{1}{E_x}\int_{-\infty}^{\infty} t|x(t)|^2 dt \qquad (s62)$$

$$\langle \omega\rangle = \frac{1}{E_x}\int_{-\infty}^{\infty} \omega|\hat{x}(\omega)|^2 d\omega \qquad (s63)$$

Where: $\hat{x}(\omega)$ is the Fourier transform of the signal, $\langle t\rangle$ and $\langle \omega\rangle$ are the average time and frequency, and E_x is the total energy of the signal:

$$E_x = \int_{-\infty}^{\infty} |x(t)|^2 dt = \int_{-\infty}^{\infty} |\hat{x}(\omega)|^2 d\omega \qquad (s64)$$

The uncertainty product of the signal $x(t)$ is defined as:

$$\mathrm{UCP}(x(t)) = \sqrt{B^2T^2} \geq \frac{1}{2} \qquad (s65)$$

UCP is an elegant way to measure the localization of the signal by computing the first and second moments in time and frequency. According to the uncertainty principle a signal cannot be at the same time arbitrarily short and achieve high concentration in frequency. The most localized signals are the Gaussians, for which UCP=1/2. Any other signals have a higher UCP. **It should be noted however that the UCP is only interpretable in the context of resolution when the distributions in time and frequency are unimodal**[23]**.**

The question is if the same UCP formalism can be used to quantify how localized a signal's TFR is. Consider the representation of the signal that has an energy density meaning, $\mathrm{TFR}_x(t,\omega)$. We first define the time and frequency marginals as:

$$\mathrm{TFR}_{x_T}(t) = \int_{-\infty}^{\infty} \mathrm{TFR}(t,\omega) d\omega \qquad (s66)$$

$$\mathrm{TFR}_{x_F}(\omega) = \int_{-\infty}^{\infty} \mathrm{TFR}(t,\omega) dt \qquad (s67)$$

The uncertainty product of the representation can be defined as:

$$\mathrm{UCP}[\mathrm{TFR}_x(t,\omega)] = \sqrt{{B_r}^2{T_r}^2} \qquad (s68)$$

were:

$$\langle t_r \rangle = \frac{1}{E_r} \int_{-\infty}^{\infty} t \, \mathrm{TFR}_{x_T}(t) dt \qquad (s69)$$

$$\langle \omega_r \rangle = \frac{1}{E_r} \int_{-\infty}^{\infty} \omega \, \mathrm{TFR}_{x_F}(\omega) d\omega \qquad (s70)$$

$${T_r}^2 = \frac{1}{E_r} \int_{-\infty}^{\infty} (t - \langle t \rangle)^2 \, \mathrm{TFR}_{x_T}(t) dt \qquad (s71)$$

$${B_r}^2 = \frac{1}{E_r} \int_{-\infty}^{\infty} (\omega - \langle \omega \rangle)^2 \, \mathrm{TFR}_{x_F}(\omega) d\omega \qquad (s72)$$

$$E_r = \int_{-\infty}^{\infty}\int_{-\infty}^{\infty} \mathrm{TFR}_x(t,\omega)dtd\omega \tag{s73}$$

Real, oscillatory signals have symmetric spectra in the frequency domain with $\langle\omega\rangle$ always 0. This particularity renders the above formalism unfit to determine how concentrated their spectra are[24]. Consider a Gaussian window $g(t)$ and one Gaussian "atom" $a(t)$ obtained by windowing a cosine wave with $g(t)$, and their power spectra (Supplementary Fig. 9a):

$$g(t) = e^{-\frac{t^2}{2\sigma^2}} \tag{s74}$$

$$a(t) = \cos(\omega_0 t)\cdot g(t) \tag{s75}$$

For both the signal $a(t)$ and the window $g(t)$ the spread in time is essentially the same (Supplementary Fig. 9b). The differences appear in the frequency domain where the window is concentrated around 0 while the atom has two "narrow" Gaussians at $\pm\omega_0$, the carrier frequency (Supplementary Fig. 9c – red traces). Intuitively, both signals are equally concentrated in frequency, but because of the symmetry, the spread of $a(t)$ is dominated by the carrier frequency:

$${B_x}^2 = \frac{(2\sigma^2{\omega_0}^2+1)e^{\sigma^2{\omega_0}^2}-1}{2\sigma^2 e^{\sigma^2{\omega_0}^2}-2\sigma^2} \tag{s76}$$

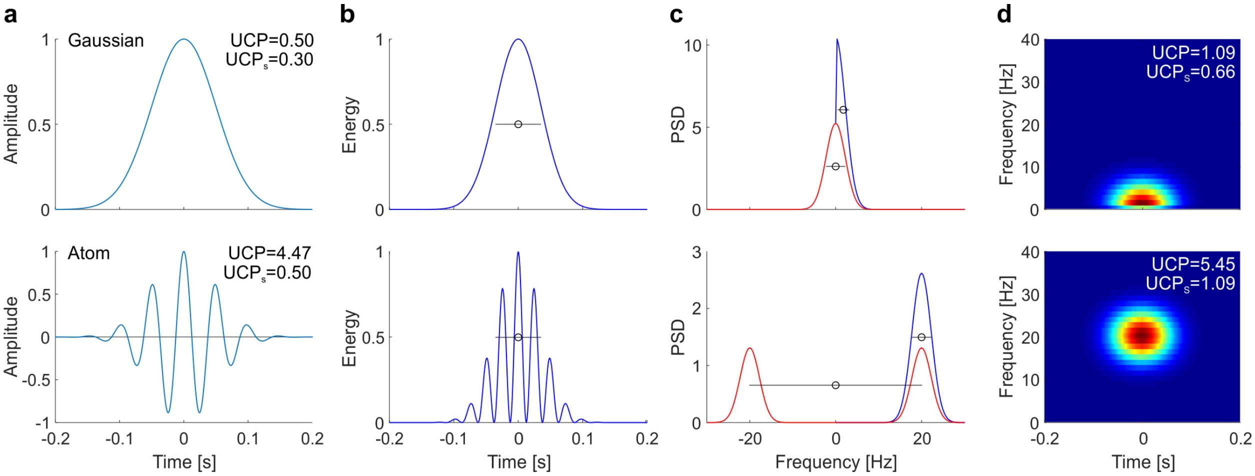

Supplementary Fig. 9 Uncertainty product. a The Gaussian window (top) and atom (bottom). **b** Energy of the Gaussian window and atom in time. The black circle is the mean

time, $\langle t \rangle$, and the black bars depict the spread in time, $\pm T$. **c** Power spectral density of the signals is shown with blue and red for the single and double-sided spectra respectively. Like for the time plots, the mean frequency of the signal, $\langle \omega \rangle$, and the spread, $\pm B$, are show in black. **d** The spectrogram of the two signals as an example of a one-sided representation.

Provided that we restrict to only the positive side of the spectrum (Supplementary Fig. 9c – blue traces), the spread in frequency for $a(t)$ is freed from the influence of the carrier and the UCP formalism seems suitable (see also Folland and Sitaram, 1997[25]). Here we use the single sided uncertainty product, UCPS, where we restrict the frequency domain only to the positive side of the spectrum:

$$\text{UCPS}[x(t)] = \text{UCP}|_{\hat{x}(\omega)=0,\ \ \omega<0} \qquad (s77)$$

$$\text{UCPS}[\text{TFR}_x(t,\omega)] = \text{UCP}|_{\text{TFR}_x(t,\omega)=0,\ \ \omega<0} \qquad (s78)$$

Since the spectrum of the atom $a(t)$ is a translated version of the spectrum of the Gaussian $g(t)$, we expect its UCPS to be 1/2 which is indeed the case. However, the single sided uncertainty product does not produce correct values for the window $g(t)$, UCPS_g=0.30 below the 1/2 theoretical value[11]. The reason is visible in (Supplementary Fig. 9c) where the single side representation of $g(t)$, is narrowed compared to the double side spectrum. The same observation can be made on the signal representations (Supplementary Fig. 9d). The UCP of the signal representation that relies on the window function is above 1 due to spreading induced by the window[26]. $\text{UCPS}_{\text{TFR}a}$=1.09 close to the expected value for a concentrated signal, but for the window g, $\text{UCPS}_{\text{TFR}g}$ is only 0.66.

Thus, the single side formalism reveals the expected localization for signal representations that have components above DC. In practice this is not a real impediment, since in most situations the DC is removed at acquisition or in the preprocessing stages.

Next, we used the UCPS formalism to quantify the localization of the *superlets* scalogram representation for mono-component signals composed of atoms and single sine packets. We computed the UCPS of the signals with eq. (s60-s65) and then compared it to the UCPS' of scalogram representations using the SLT (order 8) and each individual wavelet that was part of the SLT set (Supplementary Fig. 10). The c_1 parameter was

computed such that the shortest wavelet's Morlet standard deviation would match the standard deviation of the atom's underlying Gaussian window.

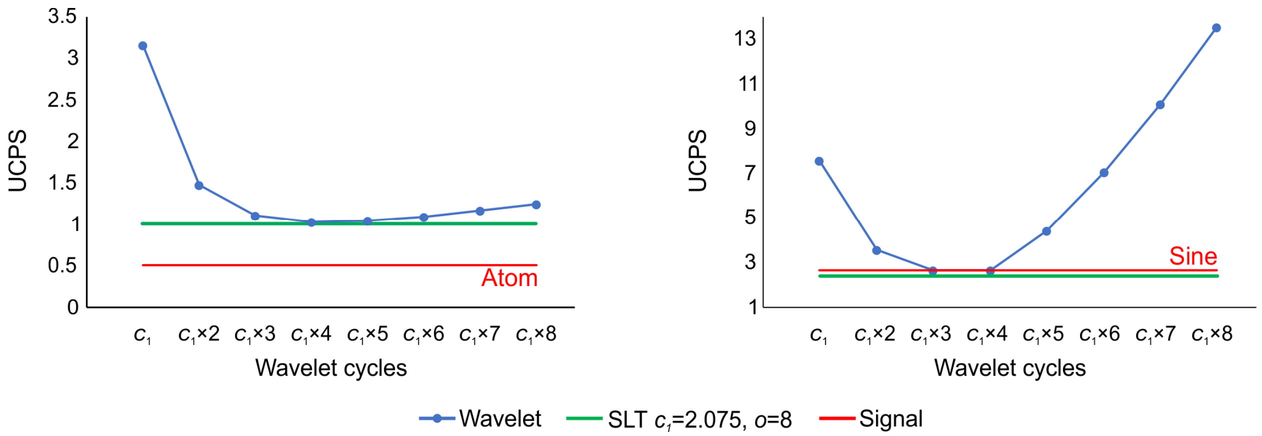

Supplementary Fig. 10 Single sided uncertainty product. The UCPS is computed on two different signals (atom and sine – red), and on the representations produced with the SLT (green) or the individual wavelets that compose the SLT (blue).

Results indicate that the UCPS of *superlet* scalograms was always at or below the UCPS of scalograms of the composing wavelets. In addition, none of the representations could reach the UCPS of the atom, which is the most possibly localized signal. For the sine signal, the UCPS of the *superlets* is actually a bit below the UCPS of the signal.

Marginals of high-resolution representations

The UCP relies on the marginals of the TFR/TSR to characterize the "quality" of a representation. We would like to argue, as has also been suggested before, that marginals are in fact not very useful for the evaluation of a certain representation's capabilities. To investigate this issue, we computed the TFRs/TSRs for a series of simple to complex signals (Supplementary Fig. 11).

The Wigner-Ville Distribution (WVD) based methods (Supplementary Fig. 11, top 3 rows) perform very well on simple signals, like the Gaussian atom, and clearly outperform the ASLT and MMCE. On the chirp signal, only the WVD performs well, while its smoothed variants (Choi-Williams – CW; Born-Jordan – BJ) actually perform worse that the ASLT and

MMCE. On more complex, multi-component signals, like the example from Fig. 3 in Main Text, the WVD, CW, and BJ underperform, with poor time resolution and being plagued by multiple cross-terms, in time, frequency, or both (Supplementary Fig. 11, "Neighbors").

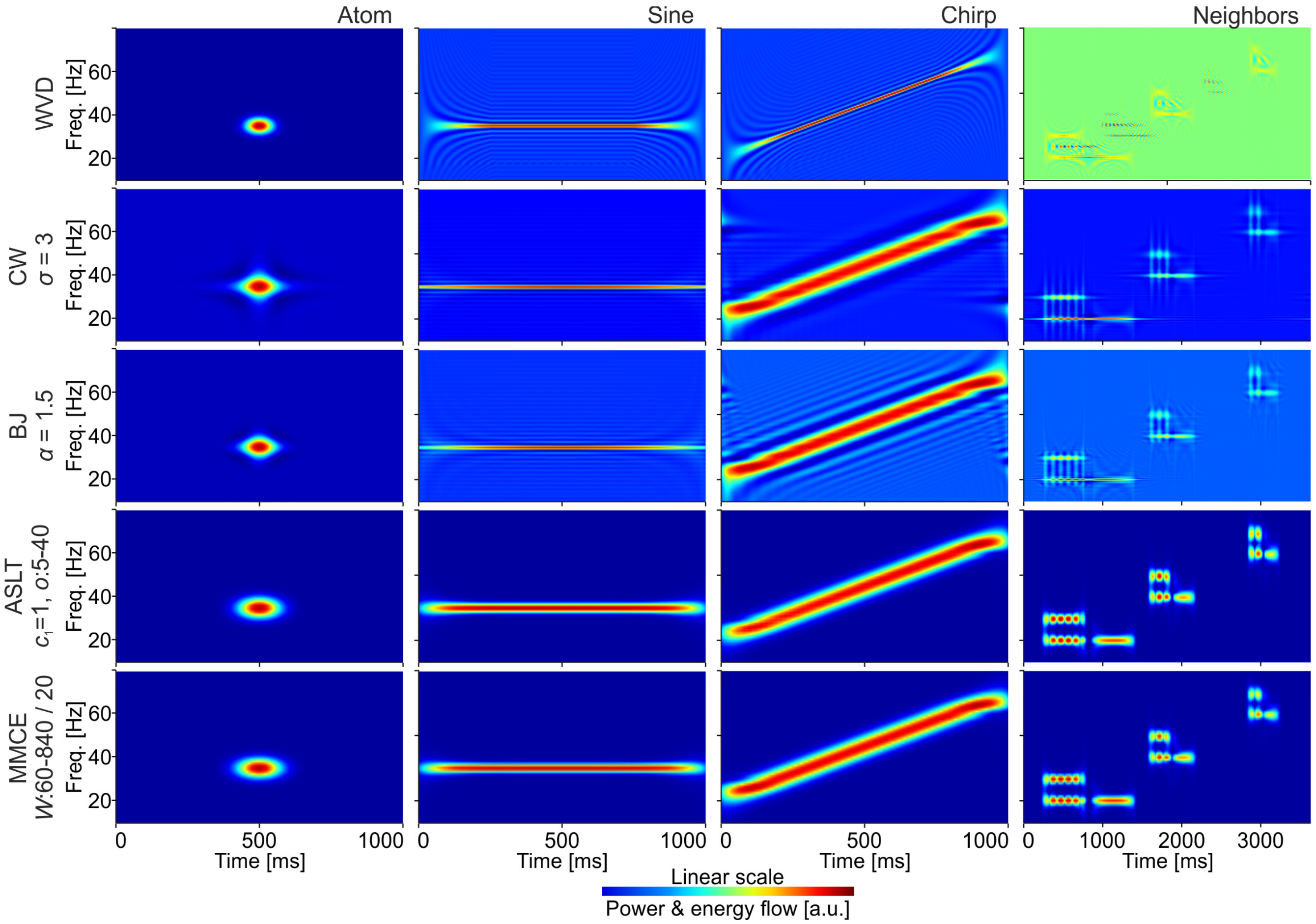

Supplementary Fig. 11 TFR/TSR of various types of signals, with different methods. WVD (Wigner-Ville Distribution), CW (Choi-Williams), BJ (Born-Jordan), ASLT, and MMCE. Absolute power / energy flow shown (linear scale).

By contrast, the ASLT and MMCE do an excellent job, with clearly superior time and frequency resolution. It should be noted that here we opted for ASLT due to the large frequency range. For smaller frequency ranges the SLT provides a sharper, non-diluted representation than both the ASLT and the MMCE. For brain signals, we recommend using the SLT for the 30-80 Hz range (gamma band).

For each of the representations in Supplementary Fig. 11 we computed the time and frequency marginals (Supplementary Fig. 12).

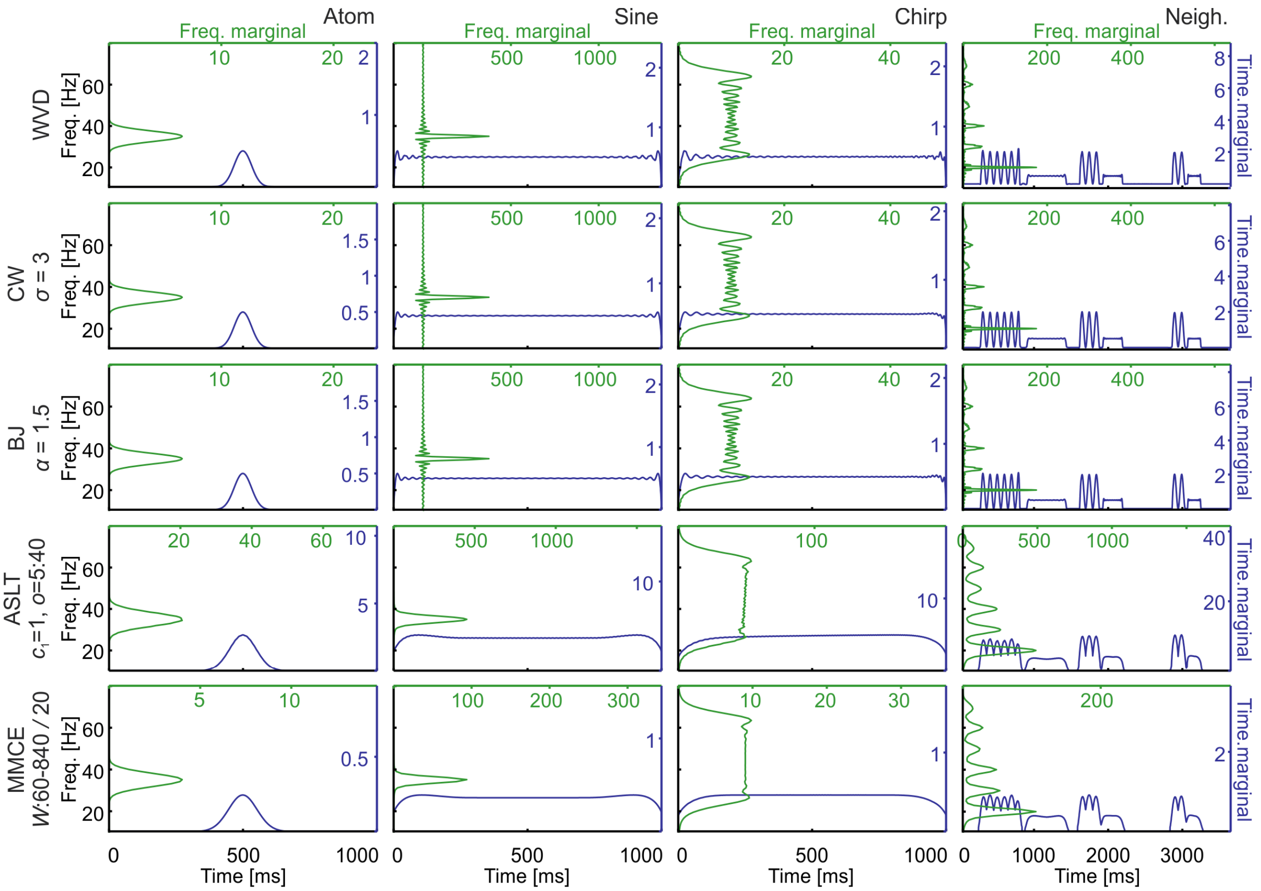

Supplementary Fig. 12 Time (blue) and frequency (green) marginals of different representations on various signals. Marginals were computed on the representations from Supplementary Fig. 11.

A first observation is that the WVD and its directionally smoothed variants (CW and BJ) conserve the marginals, i.e. the integrated marginals correctly provide the energy of the signal. The next best is the MMCE, which is based on the spectrogram. By contrast, in the ASLT the total energy estimate is higher than in reality (note the scales). One should note however that the conservation of marginals is not a purpose in itself[2], especially when the method is used as a detection tool. Many very useful representations, like the scalogram, do not preserve marginals[7]. Second, for the more complex signals, like the time-frequency

"neighbors", the marginals are deceiving, suggesting that the WVD, CW, and BJ are the sharpest representations (Supplementary Fig. 12, right), when in fact they are the poorest (Supplementary Fig. 11, right). The reason is that various cross-terms, which plague the representation, sum up in ways that either cancel out or sum up, such that the marginals falsely indicate a more accurate representation than it is in reality.

In the Main Text, we discuss why the UCP is not equivalent to resolution and therefore the marginals may prove useless to determine a method's resolution. In general, moment-based measures, like the UCP, do not truly measure signal complexity or information content[27]. Several other techniques to measure a representation's resolution have been proposed[23,28].

V. Gallery of examples on single trial electrophysiology data

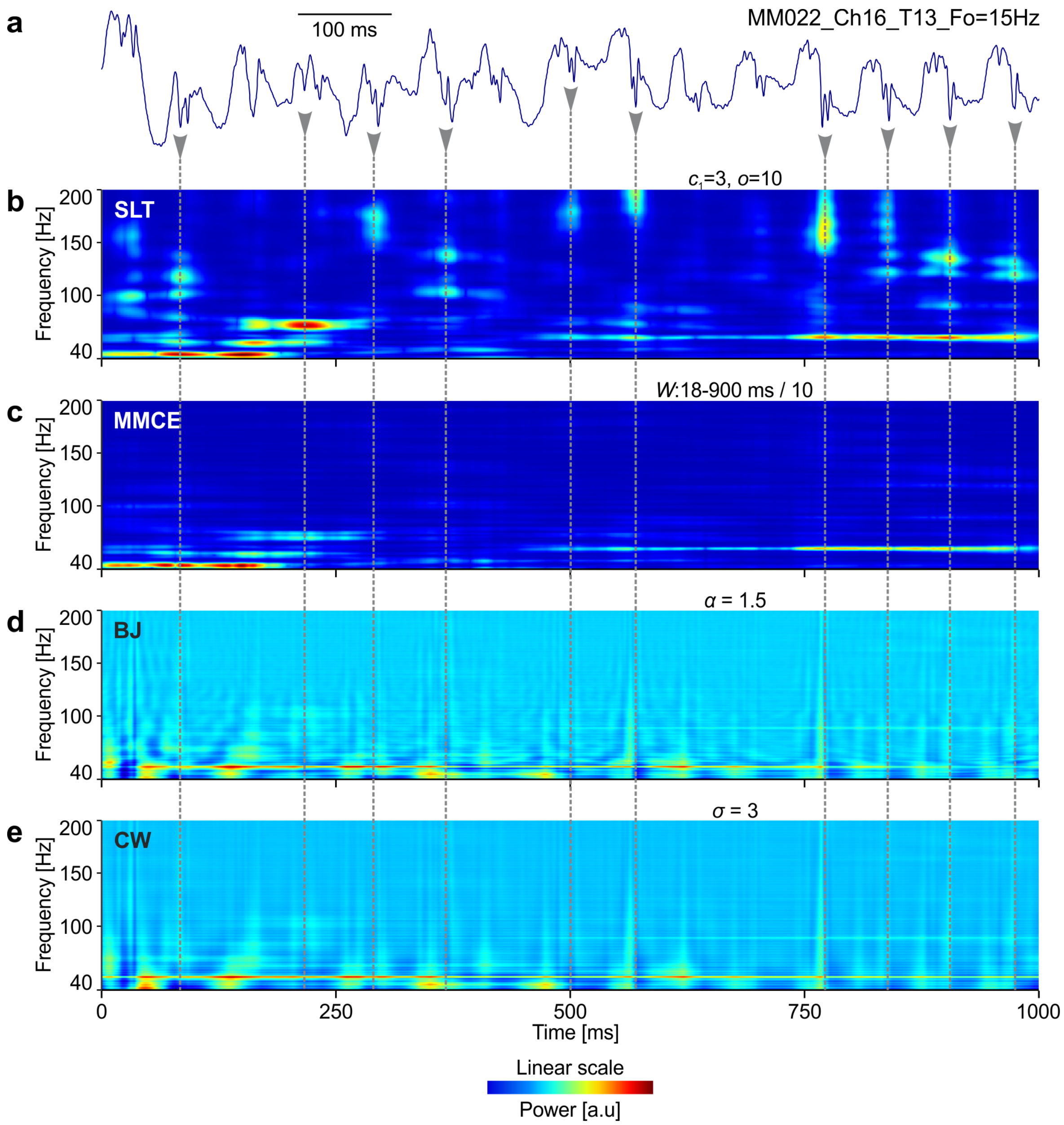

Supplementary Fig. 13 Time-frequency representations of a single LFP trial recorded with 15 Hz optogenetic stimulation. The MMCE parameters were matched to those of the SLT. Absolute power / energy flow shown (linear scale).

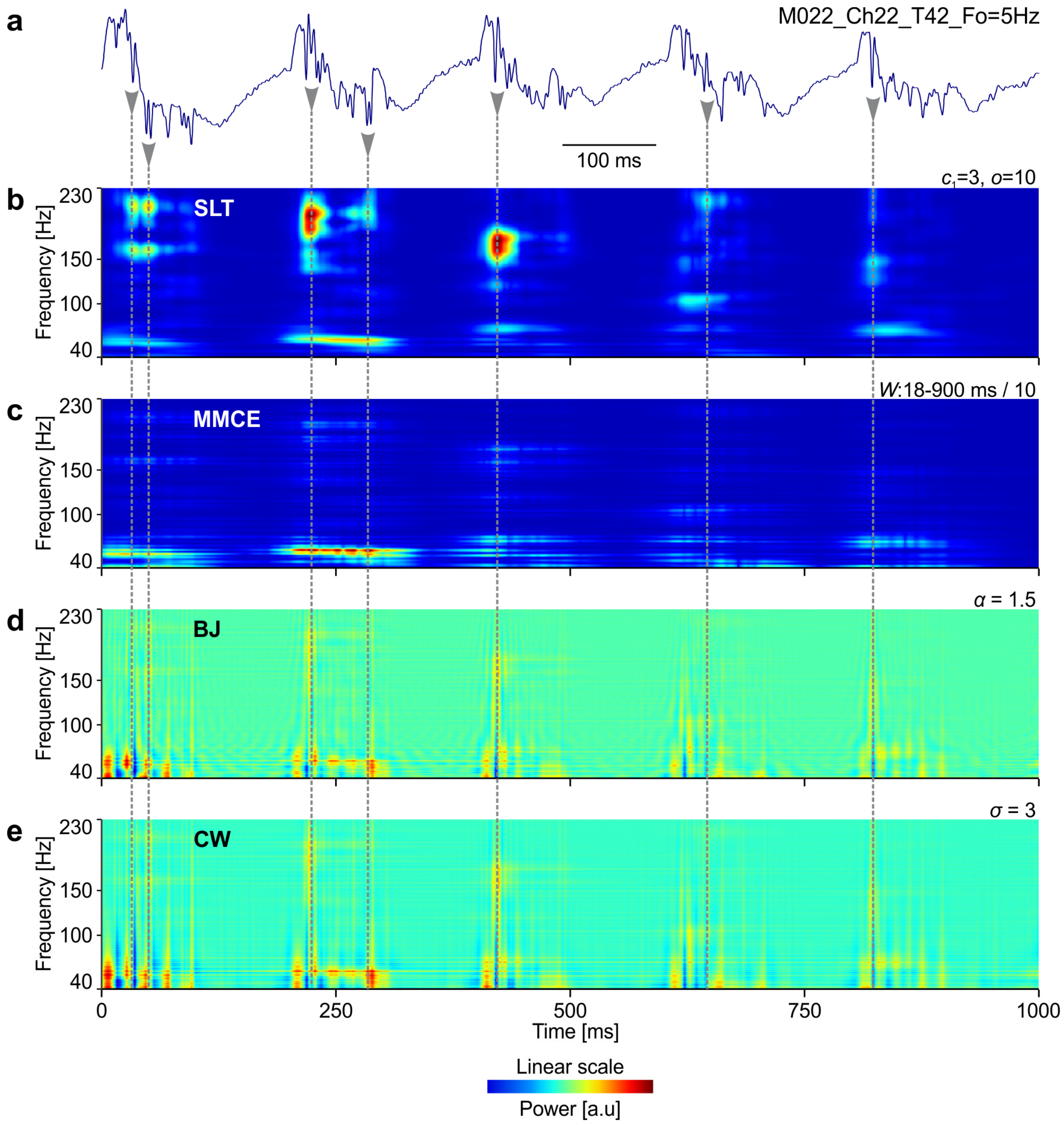

Supplementary Fig. 14 Time-frequency representations of a single LFP trial recorded with 5 Hz optogenetic stimulation. The MMCE parameters were matched to those of the SLT. Absolute power / energy flow shown (linear scale).

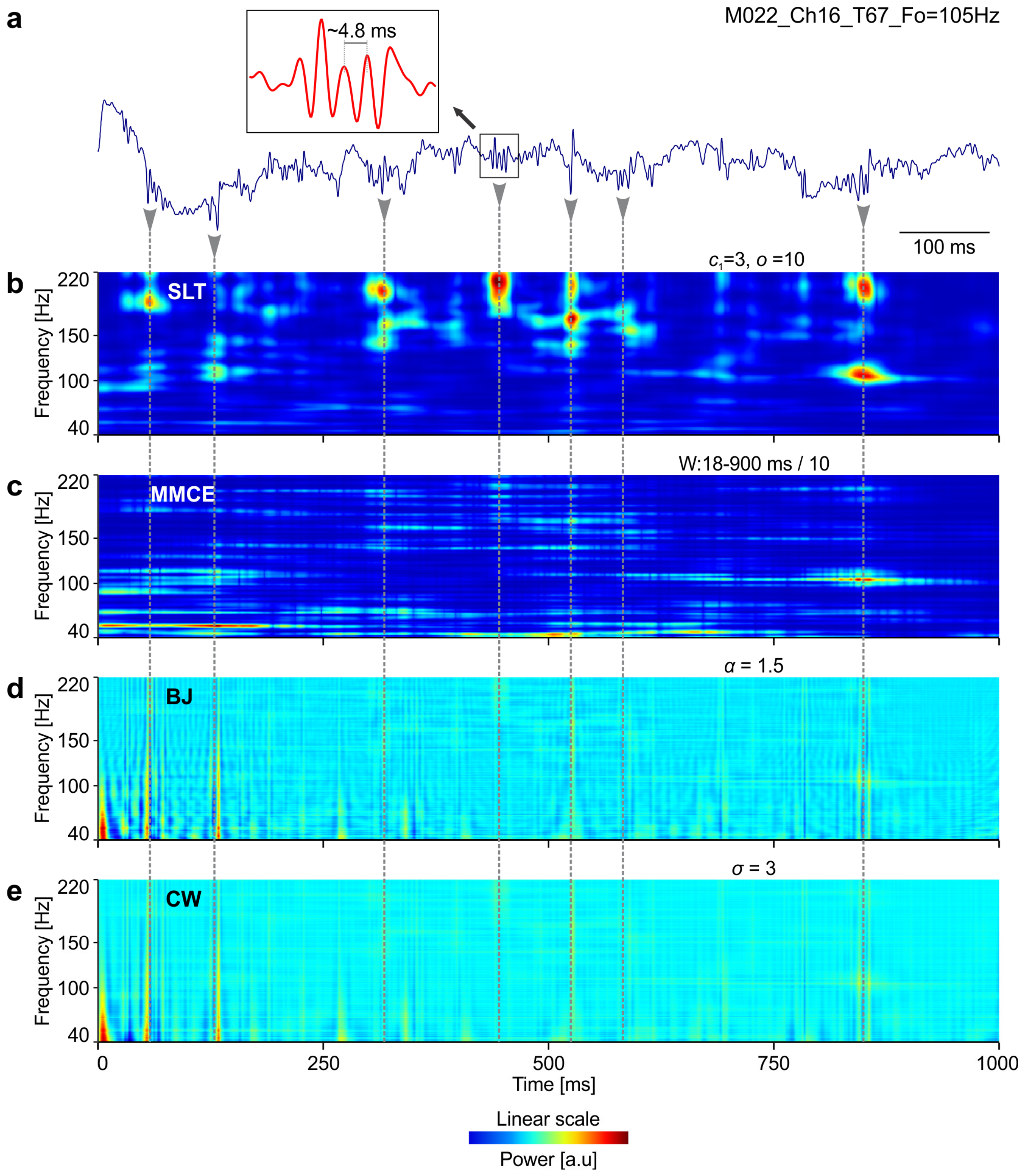

Supplementary Fig. 15 Time-frequency representations of a single LFP trial recorded with 105 Hz optogenetic stimulation. The MMCE parameters were matched to those of the SLT. The inset shows a 210 Hz transient burst. Absolute power / energy flow shown (linear scale).

VI. Supplementary Information references

1. Cohen, L. *Time-frequency Analysis*. (Prentice Hall PTR, 1995).
2. Boashash, B. *Time-Frequency Signal Analysis and Processing: A Comprehensive Reference*. (Academic Press, 2015).
3. Auger, F. & Flandrin, P. Improving the readability of time-frequency and time-scale representations by the reassignment method. *IEEE Transactions on Signal Processing* **43**, 1068–1089 (1995).
4. Cohen, L. & Posch, T. Positive time-frequency distribution functions. *IEEE Transactions on Acoustics, Speech, and Signal Processing* **33**, 31–38 (1985).
5. Flandrin, P. & Rioul, O. Affine smoothing of the Wigner-Ville distribution. in *International Conference on Acoustics, Speech, and Signal Processing* 2455–2458 vol.5 (1990). doi:10.1109/ICASSP.1990.116088.
6. Grossmann, A. & Morlet, J. Decomposition of Hardy Functions into Square Integrable Wavelets of Constant Shape. *SIAM J. Math. Anal.* **15**, 723–736 (1984).
7. Rioul, O. & Flandrin, P. Time-scale energy distributions: a general class extending wavelet transforms. *IEEE Transactions on Signal Processing* **40**, 1746–1757 (1992).
8. Grossmann, A., Kronland-Martinet, R. & Morlet, J. Reading and Understanding Continuous Wavelet Transforms. in *Wavelets* (eds. Combes, J.-M., Grossmann, A. & Tchamitchian, P.) 2–20 (Springer, 1990). doi:10.1007/978-3-642-75988-8_1.
9. Torrence, C. & Compo, G. P. A Practical Guide to Wavelet Analysis. *Bulletin of the American Meteorological Society* **79**, 61–78 (1998).
10. Farge, M. Wavelet transforms and their applications to turbulence. *Annual review of fluid mechanics* **24**, 395–458 (1992).
11. Cohen, L. The Uncertainty Principle for the Short-Time Fourier Transform and Wavelet Transform. in *Wavelet Transforms and Time-Frequency Signal Analysis* (ed. Debnath, L.) 217–232 (Birkhäuser, 2001). doi:10.1007/978-1-4612-0137-3_8.

12. Zhong, S. & Mallat, S. Characterization of Signals from Multiscale Edges. *IEEE Transactions on Pattern Analysis & Machine Intelligence* **14**, 710–732 (1992).

13. Delprat, N. *et al.* Asymptotic wavelet and Gabor analysis: extraction of instantaneous frequencies. *IEEE Transactions on Information Theory* **38**, 644–664 (1992).

14. Carmona, R. A., Hwang, W. L. & Torresani, B. Characterization of signals by the ridges of their wavelet transforms. *IEEE Transactions on Signal Processing* **45**, 2586–2590 (1997).

15. Mallat, S. *A Wavelet Tour of Signal Processing: The Sparse Way*. (Academic Press, 2008).

16. Lilly, J. M. Element analysis: a wavelet-based method for analysing time-localized events in noisy time series. *Proc Math Phys Eng Sci* **473**, (2017).

17. Liu, L., Hsu, H. & Grafarend, E. W. Normal Morlet wavelet transform and its application to the Earth's polar motion. *Journal of Geophysical Research: Solid Earth* **112**, (2007).

18. Ouillon, G., Sornette, D. & Castaing, C. Organisation of joints and faults from 1-cm to 100-km scales revealed by optimized anisotropic wavelet coefficient method and multifractal analysis. *Nonlinear Processes in Geophysics* **2**, 158–177 (1995).

19. Rizzo, R. E., Healy, D., Farrell, N. J. & Heap, M. J. Riding the Right Wavelet: Quantifying Scale Transitions in Fractured Rocks. *Geophysical Research Letters* **44**, 11,808-11,815 (2017).

20. Audet, P. & Mareschal, J.-C. Wavelet analysis of the coherence between Bouguer gravity and topography: application to the elastic thickness anisotropy in the Canadian Shield. *Geophys J Int* **168**, 287–298 (2007).

21. Mathworks. cwt.m normalization. https://www.mathworks.com/matlabcentral/answers/339748-cwt-m-normalization.

22. Wilson, R., Calway, A. D. & Pearson, E. R. S. A generalized wavelet transform for Fourier analysis: the multiresolution Fourier transform and its application to image and audio signal analysis. *IEEE Transactions on Information Theory* **38**, 674–690 (1992).

23. Stanković, L. A measure of some time–frequency distributions concentration. *Signal Processing* **81**, 621–631 (2001).

24. Dodonov, V. V. & Dodonov, A. V. Energy–time and frequency–time uncertainty relations: exact inequalities. *Phys. Scr.* **90**, 074049 (2015).

25. Folland, G. B. & Sitaram, A. The uncertainty principle: A mathematical survey. *The Journal of Fourier Analysis and Applications* **3**, 207–238 (1997).

26. Cohen, L. Uncertainty principles of the short-time Fourier transform. in *Advanced Signal Processing Algorithms* vol. 2563 80–90 (International Society for Optics and Photonics, 1995).

27. Flandrin, P., Baraniuk, R. G. & Michel, O. Time-frequency complexity and information. in *Proceedings of ICASSP '94. IEEE International Conference on Acoustics, Speech and Signal Processing* vol. iii III/329-III/332 vol.3 (1994).

28. Boashash, B. & Sucic, V. Resolution measure criteria for the objective assessment of the performance of quadratic time-frequency distributions. *IEEE Transactions on Signal Processing* **51**, 1253–1263 (2003).
