# Supplementary material for: Time-frequency super-resolution with superlets
Source: Nat Commun. 2021 Jan 12;12:337. doi: 10.1038/s41467-020-20539-9 (PMC7803992; doi:10.1038/s41467-020-20539-9)
Supplement: Supplementary file 3 — Reporting Summary [file 41467_2020_20539_MOESM3_ESM.pdf]

nature research

Corresponding author(s): Raul C. Muresan

Last updated by author(s): Nov 22, 2020

Reporting Summary

Nature Research wishes to improve the reproducibility of the work that we publish. This form provides structure for consistency and transparency in reporting. For further information on Nature Research policies, see Authors & Referees and the Editorial Policy Checklist.

Statistics

For all statistical analyses, confirm that the following items are present in the figure legend, table legend, main text, or Methods section.

n/a	Confirmed	
☒	☐	The exact sample size (*n*) for each experimental group/condition, given as a discrete number and unit of measurement
☒	☐	A statement on whether measurements were taken from distinct samples or whether the same sample was measured repeatedly
☒	☐	The statistical test(s) used AND whether they are one- or two-sided<br>*Only common tests should be described solely by name; describe more complex techniques in the Methods section.*
☒	☐	A description of all covariates tested
☒	☐	A description of any assumptions or corrections, such as tests of normality and adjustment for multiple comparisons
☐	☒	A full description of the statistical parameters including central tendency (e.g. means) or other basic estimates (e.g. regression coefficient) AND variation (e.g. standard deviation) or associated estimates of uncertainty (e.g. confidence intervals)
☒	☐	For null hypothesis testing, the test statistic (e.g. *F*, *t*, *r*) with confidence intervals, effect sizes, degrees of freedom and *P* value noted<br>*Give P values as exact values whenever suitable.*
☒	☐	For Bayesian analysis, information on the choice of priors and Markov chain Monte Carlo settings
☒	☐	For hierarchical and complex designs, identification of the appropriate level for tests and full reporting of outcomes
☒	☐	Estimates of effect sizes (e.g. Cohen's *d*, Pearson's *r*), indicating how they were calculated

Our web collection on statistics for biologists contains articles on many of the points above.

Software and code

Policy information about availability of computer code

Data collection	Data was collected using the custom Biosemi EEG recording software ActiView for Windows version 6.05 and the custom Multichannel Systems MC_Rack version 4.6.2
Data analysis	Data was analyzed using custom software. A freely available version, written in C++ version 11 with an interface for Matlab 2018b and including code for producing artificially generated data can be found at: https://github.com/TransylvanianInstituteOfNeuroscience/Superlets.

For manuscripts utilizing custom algorithms or software that are central to the research but not yet described in published literature, software must be made available to editors/reviewers. We strongly encourage code deposition in a community repository (e.g. GitHub). See the Nature Research guidelines for submitting code & software for further information.

Data

Policy information about availability of data

All manuscripts must include a data availability statement. This statement should provide the following information, where applicable:

- Accession codes, unique identifiers, or web links for publicly available datasets
- A list of figures that have associated raw data
- A description of any restrictions on data availability

EEG and electrophysiology datasets analyzed for the present study are available from the corresponding author on reasonable request. EEG dataset codes are: Dots_30_001 (Fig. 4a-b and 8a-c) and Dots_30_002 (Fig. 6a-b and 7). In vivo electrophysiology codes are: M017_002 (Fig. 4c-e, 5, 6c-e, and 8d-f) and M022_004 (Fig. 10).

Field-specific reporting

Please select the one below that is the best fit for your research. If you are not sure, read the appropriate sections before making your selection.

☒ Life sciences ☐ Behavioural & social sciences ☐ Ecological, evolutionary & environmental sciences

For a reference copy of the document with all sections, see nature.com/documents/nr-reporting-summary-flat.pdf

Life sciences study design

All studies must disclose on these points even when the disclosure is negative.

Sample size	Sample size is irrelevant for the present study, which describes an analysis method. Data were used solely for the exemplification of the method.
Data exclusions	Not applicable
Replication	Not applicable
Randomization	Not applicable
Blinding	Not applicable

Reporting for specific materials, systems and methods

We require information from authors about some types of materials, experimental systems and methods used in many studies. Here, indicate whether each material, system or method listed is relevant to your study. If you are not sure if a list item applies to your research, read the appropriate section before selecting a response.

Materials & experimental systems

n/a	Involved in the study	
☒	☐	Antibodies
☒	☐	Eukaryotic cell lines
☒	☐	Palaeontology
☐	☒	Animals and other organisms
☐	☒	Human research participants
☒	☐	Clinical data

Methods

n/a	Involved in the study	
☒	☐	ChIP-seq
☒	☐	Flow cytometry
☒	☐	MRI-based neuroimaging

Animals and other organisms

Policy information about studies involving animals; ARRIVE guidelines recommended for reporting animal research

Laboratory animals	Mus musculus, C57BL/6, Emx-Cre-GCaMP3, 8 months, female; Thy1-ChR-YFP, 10 months, female. Mice were housed in a controlled environment, with temperature in the range of 21-23°C and 50-60% humidity, with a dark/light cycle of 12/12 hours.
Wild animals	No wild animals were used in the study
Field-collected samples	No field-collected samples were used in the study
Ethics oversight	Experiments were approved by the Local Ethics Committee (3/CE/02.11.2018) and the National Veterinary Authority (ANSVSA; 147/04.12.2018).

Note that full information on the approval of the study protocol must also be provided in the manuscript.

Human research participants

Policy information about studies involving human research participants

Population characteristics	Healthy human volunteers, age range 20-24, balanced male/female.
Recruitment	Participants were recruited from among undergraduate Psychology students of the Babes-Bolyai University in Cluj-Napoca. No selection bias was present.
Ethics oversight	The protocol was approved by the Local Ethics Committee (approval 1/CE/08.01.2018). Data was collected in accordance with

Ethics oversight	relevant legislation: Directive (EU) 2016/680 and Romanian Law 190/2018. All subjects gave their written informed consent before the experiment.

Note that full information on the approval of the study protocol must also be provided in the manuscript.
